# Association between Maternal Birth Weight and Prevalence of Congenital Malformations in Offspring: The Japanese Environment and Children’s Study

**DOI:** 10.3390/nu16040531

**Published:** 2024-02-14

**Authors:** Hirotaka Hamada, Noriyuki Iwama, Hasumi Tomita, Kazuma Tagami, Natsumi Kumagai, Rie Kudo, Hongxin Wang, Seiya Izumi, Zen Watanabe, Mami Ishikuro, Taku Obara, Nozomi Tatsuta, Hirohito Metoki, Masatoshi Saito, Chiharu Ota, Shinichi Kuriyama, Takahiro Arima, Nobuo Yaegashi, The Japan Environment and Children’s Study Group

**Affiliations:** 1 Department of Obstetrics and Gynecology, Tohoku University Graduate School of Medicine, 1-1, Seiryomachi, Sendai 980-8574, Miyagi, Japan; 2 Division of Molecular Epidemiology, Department of Preventive Medicine and Epidemiology, Tohoku Medical Megabank Organization, Tohoku University, 2-1, Seiryomachi, Sendai 980-8573, Miyagi, Japan; 3 Division of Molecular Epidemiology, Tohoku University Graduate School of Medicine, 2-1, Seiryomachi, Sendai 980-8575, Miyagi, Japan; 4 Environment and Genome Research Center, Tohoku University Graduate School of Medicine, 2-1, Seiryomachi, Sendai 980-8575, Miyagi, Japan; 5 Division of Public Health, Hygiene and Epidemiology, Tohoku Medical Pharmaceutical University, 1-15-1 Fukumuro, Sendai 983-8536, Miyagi, Japan; 6 Tohoku Medical Megabank Organization, Tohoku University, 2-1, Seiryomachi, Sendai 980-8573, Miyagi, Japan; 7 Department of Maternal and Fetal Therapeutics, Tohoku University Graduate School of Medicine, 1-1, Seiryomachi, Sendai 980-8574, Miyagi, Japan; 8 Department of Paediatrics, Tohoku University Graduate School of Medicine, 1-1, Seiryomachi, Sendai 980-8574, Miyagi, Japan; 9 International Research Institute of Disaster Science, Tohoku University, 468-1, Aramaki, Sendai 980-8572, Miyagi, Japan

**Keywords:** birth cohort, congenital malformations, maternal birth weight

## Abstract

Congenital malformations are functional and structural alterations in embryonic or foetal development resulting from a variety of factors including maternal health status. This study aimed to investigate the association between maternal birth weight (MBW) and the prevalence of congenital malformations in offspring using data from a nationwide birth cohort study in Japan including 103,060 pregnancies. A binary logistic regression model with adjustment for various covariates revealed that an MBW of <2500 g (low MBW) was associated with an increased risk of congenital heart disease (adjusted odds ratio: 1.388, [95% confidence interval: 1.075–1.792]), angioma (1.491 [1.079–2.059]), and inguinal hernia (1.746, [1.189–2.565]), while those with an MBW of ≥4000 g (high MBW) were associated with congenital anomalies of the urinary tract (2.194, [1.261–3.819]) and arrhythmia (1.775, [1.157–2.725]) compared with those with an MBW of 3000–3499 g. Low MBW was associated with cleft lip and/or palate (1.473, [1.052–2.064]), congenital heart disease (1.615, [1.119–2.332]), genital organs (1.648, [1.130–2.405]), hypospadias (1.804, [1.130–2.881]), and inguinal hernia (1.484, [1.189–1.851]) in male infants and CAKUT (1.619, [1.154–2.273]) in female infants, whereas high MBW was associated with congenital heart disease (1.745, [1.058–2.877]) and CAKUT (2.470, [1.350–4.517]) in male infants. The present study is the first to demonstrate a link between MBW and congenital malformations in Japanese children. While these results must be interpreted with caution, MBW should be considered a major predictor of congenital malformation risk.

## 1. Introduction

Congenital anomalies (CAs) include functional and structural alterations in embryonic or foetal development resulting from genetic, environmental, or unknown causes that originate during pregnancy, are present at birth, and cause physical or mental impairment (1). While complex genetic and environmental interactions are proposed, most CAs are of unknown aetiology. To date, approximately 50% of CAs have been linked to specific causes including genetic, socioeconomic, demographic, environmental, infectious, and maternal nutritional risk factors [1]. One of the consequences of these defects is the high death rate within the first year of life, which can contribute to long-term chronic illness and disabilities.

Epidemiological studies have linked maternal health status, such as obesity, diabetes, and nutrition, which influence the intrauterine environment, to an increased risk of CAs. A mother’s own birth weight has been reported to be associated with the risk of certain noncommunicable diseases (NCDs) in her later life, such as cardiovascular disease (CVD), diabetes mellitus (DM), and hypertensive disorders [2,3]. Recent studies have also reported that maternal birth weight (MBW) is associated with major pregnancy complications, including hypertensive disorders of pregnancy (HDP), the delivery of small-for-gestational-age infants, and gestational diabetes mellitus (GDM) [4,5,6,7,8,9].

The interplay between maternal health and offspring outcomes has long intrigued researchers, with maternal birth weight emerging as a focal point of investigation. Recent studies have highlighted the potential transgenerational effects of health status during pregnancy. Transgenerational effects were reported in the Dutch famine study, where famine exposure in utero was associated with poor health in the later life of the participants’ grandchildren [10]. Epigenetic changes are thought to be involved in shaping the health trajectory of offspring including teratogenesis, especially via the alteration of germ cell epigenome [11,12]. Of particular interest is the link between maternal birth weight and the occurrence of congenital malformations in offspring, underscoring the importance of elucidating the epigenetic mechanisms underlying intergenerational health disparities. By examining the association between maternal birth weight and the occurrence of congenital malformations in offspring, we aim to shed light on the multifaceted nature of intergenerational health effects and pave the way for targeted interventions to mitigate adverse outcomes across generations.

The Japan Environment and Children’s Study (JECS) is a nationwide prospective birth cohort study funded by the Ministry of the Environment of Japan. The JECS aims to investigate the long-term effects of exposure to chemical substances in the environment on the growth, development, incidence of disease, and change in health conditions of children, and the present study aimed to assess the association between MBW and the risk of common congenital anomalies and malformations after adjusting for potential confounding risk factors in a large nationwide birth cohort study.

## 2. Materials and Methods

### 2.1. Study Design

This prospective study used data from the JECS, a nationwide birth cohort study being conducted in Japan. The main objective of the JECS is to investigate the environmental factors associated with offspring health and development. A total of 103,060 pregnancies (and their paired partner and infant, if accessible) were recruited from 15 Regional Centres in Japan between January 2011 and March 2014. The JECS protocol was reviewed and approved by the Ministry of Environment’s Institutional Review Board on Epidemiological Studies and the Ethics Committees of all participating institutions, and written informed consent was obtained from all the participants in the JECS. Details of the study design and baseline characteristics of the JECS participants have been described previously [13,14]. Maternal information was obtained from two self-administered questionnaires, MT1 and MT2, which were collected during the first and the second or third trimester, respectively. Information on drug use during pregnancy was obtained from two interviews, ‘In-T1′ and ‘In-T2′ [15]. The study participants also answered two more questionnaires, C6m and C2y, six months and two years after delivery, respectively. The present study was based on two datasets, “jecs-ta-20190930” and “jecs-qa-20210401”, released in October 2019 and April 2021 by the Programme Office, respectively, and was integrated for further analysis, as described in the previous JECS report [16].

### 2.2. Maternal Birth Weight (MBW)

The primary exposure used in this study was MBW. Self-reported MBW was obtained using the C6m questionnaire. Based on previous studies, MBW was categorized as follows: <2500 g, 2500–2999 g, 3000–3499 g, 3500–3999 g, and ≥4000 g [7,17].

Data with improbable MBW values (<500 g or >6000 g) were excluded from further analysis [18,19].

### 2.3. Infant Congenital Malformations Selection

The primary outcome of interest in this study was congenital malformations in infants. After physicians, midwives/nurses, and/or research co-ordinators transcribed information on infant congenital malformations, the datasets on infant congenital malformations at birth (Dr0m) and one month after birth (Dr1m) were provided. The details of infant congenital malformations in the JECS have been described previously [20]. Additionally, if the caregivers answered that their infant was diagnosed as having congenital malformations in the C2y questionnaire, information on infant congenital malformations was also transcribed by physicians from medical records (namely, “Disease Data Registry”). Information on the “Disease Data Registry”, including infant congenital malformations, was provided in the dataset “jecs-qa-20210401”. If infant congenital malformations were diagnosed in either the Dr0m, the Dr1m, or the “Disease Data Registry”, the malformations were defined as positive in this study, except for umbilical hernia. The umbilical hernia was defined as positive if it was diagnosed in either the Dr0m or the “Disease Data Registry” in this study. In a previous study, congenital anomalies of the kidney and urinary tract (CAKUT) were defined as hydronephrosis, cystic renal malformations, renal agenesis, and bladder exstrophy/cloacal exstrophy [21].

Considering the sample size, we selected several eligible infant congenital malformations as outcomes to be analysed as follows: “nervous system”, “eye, ear, and face”, “cleft lip and/or cleft palate”, “congenital heart disease”, “arrhythmia”, “urinary system (CAKUT)”, “genital organs in male infants”, “limbs” of musculoskeletal system, “angioma” of skin, and “inguinal hernia”. Among “genital organs in male infants”, hypospadias and cryptorchidism/nonpalpable testis were also selected as separate outcomes.

### 2.4. Data Collection and Classification of Other Variables

The maternal age in the MT1 questionnaire was provided in the dataset. Maternal height and pre-pregnancy body weight (BW) were transcribed from medical records. If either maternal height or pre-pregnancy BW was missing, self-reported values were obtained. The pre-pregnancy body mass index (BMI) was calculated as follows: pre-pregnancy BW in kilograms divided by the height in centimetres squared. Physicians, midwives, nurses, and/or research co-ordinators transcribed information on parity, conception method, HDP, GDM, delivery week, infant birth weight, and sex from the medical records. Two parity groups were formed: primiparous and multiparous. Conception methods were classified as spontaneous pregnancy, non-assisted reproductive technology (ART), and ART. Non-ART was defined as ovulatory induction, and artificial insemination by the husband was defined as in vitro fertilisation and embryo transfer (IVF-ET), as well as intracytoplasmic sperm injection (ICSI). The medical history of maternal diseases (hypertension, type 1 diabetes, type 2 diabetes, kidney disorders, mental diseases, congenital heart diseases, uterine malformations, malformations of the urinary tract, or genital organs) was collected from the MT1 questionnaire. The medical history of kidney disease was defined as immunoglobulin A nephropathy, glomerular nephritis, and/or nephrotic syndrome. The medical history of mental diseases was also obtained, being defined as depression, anxiety disorder, schizophrenia, or dysautonomia. Data on smoking history, alcohol consumption, and marital status were obtained using the MT1 questionnaire. The choices for smoking history in the MT1 questionnaire were “Never”, “Previously did, but quit before realizing current pregnancy”, “Previously did, but quit after realizing current pregnancy”, and “Currently smoking”. In this study, the smoking status was reclassified according to the presence or absence of current smoking. Choices for alcohol consumption in the MT1 questionnaire were “Never”, “Quit drinking”, and “Continue drinking”. Alcohol consumption was classified according to the presence or absence of continued alcohol consumption. Marital status was classified as unmarried, divorced, widowed, unmarried, divorced, or widowed. Glycosylated haemoglobin (HbA1c) levels, defined by the National Glycohemoglobin Standardization Program (NGSP), were assayed at <24 weeks of gestation, using high-performance liquid chromatography (ADAMS-A1c HA-8160, Arkray, Inc., Kyoto, Japan) [22]. Information regarding the use of any drug (methimazole, selective serotonin reuptake inhibitor [SSRI], antidepressant drug except for SSRI, antianxiety, sleeping pill, antipsychotic, valproic acid, antiepileptic except for valproic acid, lithium carbonate, and other psychoactive drugs) at <12 weeks of gestation and the use of a folic acid supplement at <12 weeks of gestation were obtained from the In-T1 and In-T2 interviews. Both the maternal highest level of education and annual household income were obtained from the MT2 questionnaire. The maternal highest level of education was categorized as follows: <13 years (junior high school or high school) and ≥13 years (high school or technical junior college or technical/vocational college or associate degree or bachelor’s degree or Graduate degree [Master’s/Doctorate]). Annual household income was categorized into <4.4–5.99 and ≥6 million Japanese Yen. Information on chromosomal abnormalities or other syndromes of infants was obtained from Dr0m, Dr1m, and C2y.

### 2.5. Statistical Analysis

The continuous and categorical variables of the characteristics of study participants, including infant congenital malformations, were expressed as mean (standard deviation [SD]) and number (percentage), respectively.

The association between MBW and the prevalence of infant congenital malformations was investigated using a binary logistic regression model, and the odds ratios (ORs) were calculated. If a complete or quasi-complete separation existed, the Firth method was applied to the binary logistic regression model [23]. Participants with an MBW of 3000–3499 g were assigned to the reference category. Model 1 was defined as the crude model. Model 2 was adjusted for covariates as follows: maternal age in the MT1 questionnaire, pre-pregnancy BMI, conception method, parity (primipara or not), history of mental illness, history of maternal congenital heart disease, history of maternal uterine malformation and/or urogenital malformation, history of kidney diseases, smoking status, alcohol consumption, marital status, education level, annual income, use of any drug at <12 weeks of gestation (methimazole, SSRI, antidepressant drug except for SSRI, antianxiety, sleeping pill, antipsychotic, valproic acid, antiepileptic except for valproic acid, lithium carbonate, and other psychoactive drug), use of folic acid supplement at <12 weeks of gestation, HbA1c level at <24 weeks of gestation, and infant sex. No strong multicollinearity among covariates was confirmed using the variance inflation factor of a general linear model with infant congenital malformations as the dependent variable. As several covariates had missing data, multiple imputations using a Markov chain Monte Carlo simulation were applied. After the generation of ten datasets and their analysis, the adjusted ORs of the combined ten results were reported in the manuscript.

Stratified analysis according to infant sex was also performed to investigate differences in the association of MBW with congenital malformations between male and female infants. In the stratified analysis, infant sex was not included in Model 2.

Statistical analyses were performed using SAS version 9.4 (SAS Institute Inc., Cary, NC, USA) and R (version 4.1.2) [24].

## 3. Results

### 3.1. Characteristics of Study Participants

**Figure 1:**
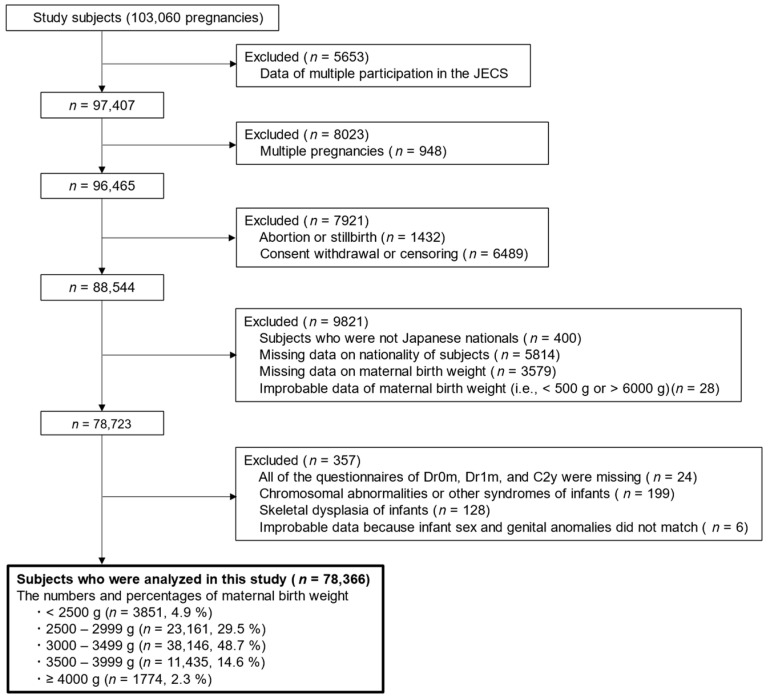
Flow chart of this study.

A flowchart of the study is shown in Figure 1. Of the 103,060 pregnancies recorded in the JECS, 5653 pregnancies with multiple participation were excluded. Participants with multiple pregnancies (*n* = 8023), abortion or stillbirth (*n* = 1432), consent withdrawal or censoring (*n* = 6489), non-Japanese nationals (*n* = 400), missing data on the nationality of participants (*n* = 5814), missing data on MBW (*n* = 3579), and improbable data on MBW (i.e., <500 g or >6000 g) (*n* = 28) were excluded. Furthermore, all questionnaires for Dr0m, Dr1m, and C2y were missing (*n* = 24); chromosomal abnormalities or other infant syndromes (*n* = 199), skeletal dysplasia of infants (*n* = 128), and improbable data because of mismatch of infant sex and genital anomalies (*n* = 6) were also excluded. Finally, 78,366 mothers and their infants were eligible for analysis in this study. The numbers and percentages of mothers according to the category of MBW were as follows: <2500 g (*n* = 3850; 4.9%), 2500–2999 g (*n* = 23,161; 29.5%), 3000–3499 g (*n* = 38,146; 48.7%), 3500–3999 g (*n* = 11,435; 14.6%), and ≥4000 g (*n* = 1774; 2.3%), respectively.

The maternal and neonatal characteristics of the study participants are shown in Table 1. The mean (SD) maternal age at MT1 and pre-pregnancy BMI were 30.9 (5.0) years and 21.2 (3.2) kg/m^2^, respectively. The percentages of preterm deliveries and low birth weight (LBW) infants were 4.4 and 7.9%, respectively. The proportions of underweight, new-onset HDP, GDM, preterm delivery, maternal highest level of education < 13 years, unmarried, divorced, or widowed, annual household income of <4 million Japanese Yen, small for gestational age (SGA) infants, and LBW in those with MBW < 2500 g were higher than those in other categories of MBW. The proportions of participants with obese, large for gestational age (LGA) infants, and macrosomia in participants with MBW of ≥4000 g were higher than those with other categories of MBW.

### 3.2. Prevalence of Infant Congenital Malformations

The prevalence of infant congenital malformations among all participants was as follows: cleft lip and/or cleft palate (0.21%), congenital heart disease (1.36%), CAKUT (0.35%), genital organs in male infants (1.12%), limbs (0.28%), angioma (0.77%), and inguinal hernia (0.49%). As shown in Table 2, a relatively higher prevalence of infant congenital malformations was observed in infants born from mothers with an MBW <2500 g than those with other MBW categories, namely, cleft lip and/or cleft palate (0.31%), genital organs in male infants (1.63%), angioma (1.12%), and inguinal hernia (0.81%). By contrast, each prevalence of arrhythmia (0.28%) and CAKUT (0.79%) in infants born from participants with MBW of ≥4000 g was higher than those with other MBW categories. The prevalence of infant congenital heart diseases born from mothers with an MBW of <2500 g (1.79%) and ≥4000 g (1.75%) was higher than those in other MBW categories.

### 3.3. Association between MBW and Prevalence of Infant Congenital Malformations

As shown in Table 3, MBW was significantly associated with the prevalence of several infant congenital malformations, including congenital heart disease, arrhythmia, CAKUT, angioma, and inguinal hernia. Compared with participants with an MBW of 3000–3499 g, those with an MBW of <2500 g had significantly higher odds of congenital heart disease, genital organs in male infants, angioma, and inguinal hernia. In model 2, the adjusted ORs were 1.388 (95% confidence interval [CI]: 1.075–1.792) for congenital heart disease, 1.648 (95% CI: 1.130–2.405) for genital organs in male infants, 1.804 (95% CI: 1.130–2.881) for hypospadias in male infants, 1.491 (95% CI: 1.079–2.059) for angioma, and 1.746 (95% CI: 1.189–2.565) for inguinal hernia, respectively. Participants with an MBW of ≥4000 g had significantly higher odds of arrhythmia and CAKUT compared with those with an MBW of 3000–3499 g. In model 2, the adjusted ORs were 1.775 (95% CI: 1.157–2.725) for arrhythmia and 2.194 (95% CI: 1.261–3.819) for CAKUT, respectively.

### 3.4. Association between MBW and Prevalence of Infant Congenital Malformations Stratified by Infant Sex

Congenital malformations are known to have biased sex distributions. In order to investigate this, we further performed a stratified analysis according to infant sex, as shown in Table 4. Participants with an MBW of <2500 g were associated with increasing odds of cleft lip and/or cleft palate in male infants. The adjusted OR was 1.473 (95% CI: 1.052–2.064) in model 2. In female infants, the association of MBW with cleft lip and/or cleft palate was not statistically significant. The U-shaped association of MBW with congenital heart disease in male infants existed. In model 2, the adjusted ORs in an MBW of <2500 g and ≥4000 g were 1.615 (95% CI: 1.119–2.332) and 1.745 (95% CI: 1.058–2.877), respectively. By contrast, the association of MBW with congenital heart disease in female infants was not statistically significant. In both male and female infants, participants with an MBW of ≥4000 g were associated with increasing odds of arrhythmia, although the adjusted OR did not reach statistical significance in male infants. In model 2, the adjusted ORs were 1.840 (95% CI: 0.999–3.387) in male infants and 1.788 (95% CI: 1.047–3.052) in female infants, respectively. Participants with an MBW of ≥4000 g were associated with increasing odds of CAKUT in male infants (the adjusted OR was 2.470 [95% CI: 1.350–4.517] in model 2), whereas those with an MBW of <2500 g were associated with increasing odds of CAKUT in female infants (the adjusted OR was 1.619 [95% CI: 1.154–2.273] in model 2). In the analysis for the association of MBW with inguinal hernia, participants with an MBW of <2500 g had significantly higher odds of inguinal hernia in male infants compared to those with an MBW of 3000–3499 g (the adjusted OR was 1.484 [95% CI: 1.189–1.851] in model 2). However, the association between MBW and inguinal hernia in female infants was not statistically significant.

## 4. Discussion

Numerous studies have identified parental, genetic, epigenetic, and environmental risk factors for congenital anomalies in neonates and infants [1]. Meanwhile, MBW has been reported to be associated with various perinatal complications, such as GDM [5] and PTD [25]. To the best of our knowledge, this study is the first to demonstrate an association between MBW and the prevalence of congenital malformations in the offspring of the Japanese population. Low MBW was associated with congenital heart disease, angioma, and inguinal hernia, while high MBW was associated with congenital anomalies of the kidney, urinary tract, and arrhythmia. Low MBW was associated with cleft lip and/or palate, congenital heart disease, genital organs, hypospadias, and inguinal hernia in male infants and CAKUT in female infants, whereas high MBW was associated with congenital heart disease and CAKUT in male infants.

Several epidemiological studies reported that MBW is associated with the risk of CVD, DM, and hypertensive disorders later in life [2,3], as well as major pregnancy complications, including HDP and GDM [4,5,6,7,8,9], which are all conditions of the mother herself. This study is unique in that it provides evidence that there is a connection between MBW and offspring health. There have been reports that MBW is associated with the delivery of preterm or small-for-gestational-age infants and that there is no consideration given to the condition of CAs when it comes to infants’ health [7]. Our current study incorporates CAs into the picture, which may serve as a starting point for a deeper understanding of CA risk factors in the future.

Low MBW and high birth weight were associated with congenital heart defects (CHDs) in male infants. Generally, congenital malformations have a sex-biased distribution. Consistent with our findings, male infants were found to have a higher prevalence of CHD than female infants [26]. CHDs are among the most common and serious birth defects. Though several established causes of CHDs have been identified, including maternal exposure, maternal phenotypes, chromosomal abnormalities, and single-gene disorders, the causative genetic mechanisms behind CHD remain poorly understood, and more than half of CHD patients lack a genetic diagnosis [27]. Low and high MBW have been reported as risk factors for abnormal metabolic status, including obesity, gestational diabetes, and diabetes mellitus. Hyperglycaemia and micronutrient deficiency in mothers are known to relate to CHD in their offspring [28,29]. Hyperglycaemia has been well studied in both human and animal models. Hyperglycaemia during embryogenesis induces hypoxia, leading to reduced PAX3 expression, which can cause CDH [30,31]. Together with these observations, oxidative stress or epigenetic changes induced by inappropriate nutrition during pregnancy, the preconception period, or even much before pregnancy, which are related to low and high MBW, might be one of the presumed mechanisms by which MBW can cause CDH.

In the current study, the association between low MBW in both sexes and high MBW in male infants was found to be quite distinct. CAKUT constitutes 20–30% of all congenital malformations, and its prevalence has been estimated to range between three and six per 1000 births, most commonly in males. Several genes such as PAX2 and HNF1B have been identified as being related to CAKUT, and epigenetic changes in these genes are responsible for the pathogenesis of the disease [32]. Maternal factors were associated with the CAKUT scores. Maternal obesity, gestational diabetes, and diabetes mellitus are known to be risk factors for CAKUT in offspring [33,34,35,36,37]. MBW is reported to be related to risk factors of obesity, gestational diabetes, and diabetes mellitus. Maternal hyperglycaemia leads to increased oxidative stress via the production of reactive oxygen species (ROS), partly by mitochondria. The produced ROS cause membrane damage, which, in turn, activates programmed cell death via proapoptotic proteins. The upregulation of pro-apoptotic proteins leads to endoplasmic reticulum stress and cell death. Abnormal apoptosis causes malformations in the major organs during the development of a foetus [38]. Together with these observations, oxidative stress or epigenetic changes induced by inappropriate nutrition during pregnancy, the preconception period, or even before pregnancy, which are related to maternal LBW and HBW, might be one of the presumed mechanisms by which MBW causes CAKUT.

Preterm infants are at risk of inguinal hernia and angioma [39,40]. Both inguinal hernias and angiomas occur in up to four percent of children and are among the most common indications for paediatric surgery [40,41]. Low MBW, sometimes caused by preterm birth, is also associated with inguinal hernias in male offspring and angiomas in all infants. Interestingly, the renin-angiotensin system (RAS) may play a role in endothelial cell proliferation in haemangiomas. Pregnant women born with LBWs have an increased risk of hypertensive disorders during pregnancy, which contributes to the risk of developing chronic hypertension in infants, in which the RAS is highly involved [42]. In line with these insights, maternal LBW may increase the risk of inguinal hernia or angioma in offspring via impaired RAS.

Viewing maternal birth weight as reflective of the intrauterine environment, the results of the current study embody the aspect of transgenerational effects. Such effects have been documented in both human and animal studies. Notably, a series of reports from the Dutch famine demonstrate transgenerational effects on the health status of grandchildren [10,43]. It is worth noting that there appears to be a sex-specific effect; associations were observed in the paternal lineage but not in the maternal lineage [43,44]. The underlying molecular mechanisms, focusing on germline cells, have been extensively studied in animal models, indicating the involvement of epigenetic modifications in transgenerational inheritance [45,46]. From the results of the current study, we could speculate that maternal inadequate birth weight, potentially resulting from certain environmental exposures during pregnancy or infancy, could lead to modifications in the epigenetic status of the oocyte. These modifications may have implications for embryonic teratogenesis, potentially resulting in the development of congenital abnormalities.

In summary, there is indirect evidence supporting the association between MBW and congenital anomalies in offspring; however, further analysis is required to unravel the molecular mechanism.

This study has several strengths. First, the study participants were recruited from a wide geographical area of Japan. Therefore, the external validity of the results of this study was high. Second, this study considered many variables, including maternal physiological and socioeconomic factors, in its statistical analysis. However, this study also has several limitations. Information on MBW was obtained from a self-reported questionnaire; therefore, it may have been misclassified. Notably, birth weights collected from medical records and self-reports were comparable [47]. Hence, we believe that this limitation did not strongly affect our findings. Second, this was a single-ethnicity study. The genetic background is homogeneous, which enables a reduction in genetic variation; however, it may not be adaptable to other ethnicities. Further studies involving different ethnicities are required to confirm our findings.

## 5. Conclusions

MBW should be considered as one of the major parameters to be collected at the first visit to obstetric clinics along with past medical history, familial history, and maternal parameters, especially birth weight, for recognising the risk of congenital malformations in offspring. However, the results of the current study must be interpreted with caution, as MBW and infants’ congenital malformations remain an association but not a direct cause. Future studies are required to unravel the mechanism of the relationship between MBW and the risk of congenital malformations of the offspring so that appropriate intervention can be provided to patients to reduce the risk of giving birth to offspring with congenital malformations.

## Figures and Tables

**Table 1:** Characteristics of study participants.

Variables	All Participants (*n* = 78,366)	Participants According to Maternal Birth Weight				
		<2500 g (*n* = 3850)	2500– 2999 g (*n* = 23,161)	3000– 3499 g (*n* = 38,146)	3500– 3999 g (*n* = 11,435)	≥4000 g (*n* = 1774)
**Maternal age in the MT1 questionnaire, years**	**30.9 (5.0)**	**30.6 (5.2)**	**30.7 (5.0)**	**31.0 (4.9)**	**31.2 (4.9)**	**31.5 (4.8)**
**Category of maternal age in the MT1 questionnaire, *n* (%)**						
<25 years	7897 (10.1)	498 (12.9)	2631 (11.4)	3653 (9.6)	976 (8.5)	139 (7.8)
25–29.9 years	22,832 (29.1)	1118 (29.0)	6971 (30.1)	11,005 (28.8)	3270 (28.6)	468 (26.4)
30–34.9 years	27,679 (35.3)	1298 (33.7)	7949 (34.3)	13,727 (36.0)	4054 (35.5)	651 (36.7)
≥35 years	19,480 (24.9)	905 (23.5)	5478 (23.7)	9530 (25.0)	3061 (26.8)	506 (28.5)
Missing	478 (0.6)	31 (0.8)	132 (0.6)	231 (0.6)	74 (0.6)	10 (0.6)
**Pre-pregnancy BMI, kg/m^2^**	21.2 (3.2)	20.9 (3.3)	20.9 (3.2)	21.2 (3.2)	21.5 (3.3)	22.0 (3.6)
**Category of pre-pregnancy BMI, *n* (%)**						
Underweight (<18.5 kg/m^2^)	12,733 (16.2)	790 (20.5)	4370 (18.9)	5973 (15.7)	1443 (12.6)	157 (8.9)
Normal range (18.5–24.9 kg/m^2^)	57,638 (73.5)	2682 (69.7)	16,664 (71.9)	28,251 (74.1)	8686 (76.0)	1355 (76.4)
Obese (≥25.0 kg/m^2^)	7936 (10.1)	373 (9.7)	2105 (9.1)	3901 (10.2)	1297 (11.3)	260 (14.7)
Missing	59 (0.1)	5 (0.1)	22 (0.1)	21 (0.1)	9 (0.1)	2 (0.1)
**Parity, *n* (%)**						
Primipara	33,188 (42.3)	1742 (45.2)	9997 (43.2)	15,854 (41.6)	4888 (42.7)	707 (39.9)
Multipara	43,210 (55.1)	2015 (52.3)	12,539 (54.1)	21,364 (56.0)	6263 (54.8)	1029 (58.0)
Missing	1968 (2.5)	93 (2.4)	625 (2.7)	928 (2.4)	284 (2.5)	38 (2.1)
**Conception method, *n* (%)**						
Spontaneous pregnancy	72,566 (92.6)	3567 (92.6)	21,492 (92.8)	35,385 (92.8)	10,487 (91.7)	1635 (92.2)
Non-ART	2984 (3.8)	147 (3.8)	860 (3.7)	1423 (3.7)	490 (4.3)	64 (3.6)
ART	2446 (3.1)	111 (2.9)	701 (3.0)	1159 (3.0)	408 (3.6)	67 (3.8)
Missing	370 (0.5)	25 (0.6)	108 (0.5)	179 (0.5)	50 (0.4)	8 (0.5)
**History of hypertension, *n* (%)**						
Yes	355 (0.5)	29 (0.8)	119 (0.5)	153 (0.4)	46 (0.4)	8 (0.5)
No	77,536 (98.9)	3790 (98.4)	22,910 (98.9)	37,763 (99.0)	11,317 (99.0)	1756 (99.0)
Missing	475 (0.6)	31 (0.8)	132 (0.6)	230 (0.6)	72 (0.6)	10 (0.6)
**History of type 1 diabetes, *n* (%)**						
Yes	61 (0.1)	3 (0.1)	19 (0.1)	30 (0.1)	7 (0.1)	2 (0.1)
No	77,830 (99.3)	3816 (99.1)	23,010 (99.3)	37,886 (99.3)	11,356 (99.3)	1762 (99.3)
Missing	475 (0.6)	31 (0.8)	132 (0.6)	230 (0.6)	72 (0.6)	10 (0.6)
**History of type 2 diabetes, *n* (%)**						
Yes	96 (0.1)	8 (0.2)	31 (0.1)	40 (0.1)	12 (0.1)	5 (0.3)
No	77,795 (99.3)	3811 (99.0)	22,998 (99.3)	37,876 (99.3)	11,351 (99.3)	1759 (99.2)
Missing	475 (0.6)	31 (0.8)	132 (0.6)	230 (0.6)	72 (0.6)	10 (0.6)
**HbA1c (NGSP) level at <24 weeks of** **gestation, %**	5.2 (0.3)	5.2 (0.3)	5.2 (0.3)	5.2 (0.3)	5.2 (0.3)	5.2 (0.3)
**History of kidney disorder, *n* (%)**						
Yes	341 (0.4)	23 (0.6)	102 (0.4)	150 (0.4)	55 (0.5)	11 (0.6)
No	77,550 (99.0)	3796 (98.6)	22,927 (99.0)	37,766 (99.0)	11,308 (98.9)	1753 (98.8)
Missing	475 (0.6)	31 (0.8)	132 (0.6)	230 (0.6)	72 (0.6)	10 (0.6)
**History of mental diseases, *n* (%)**						
Yes	5961 (7.6)	323 (8.4)	1762 (7.6)	2849 (7.5)	887 (7.8)	140 (7.9)
No	71,930 (91.8)	3496 (90.8)	21,267 (91.8)	35,067 (91.9)	10,476 (91.6)	1624 (91.5)
Missing	475 (0.6)	31 (0.8)	132 (0.6)	230 (0.6)	72 (0.6)	10 (0.6)
**History of congenital heart diseases, *n* (%)**						
Yes	259 (0.3)	22 (0.6)	79 (0.3)	109 (0.3)	44 (0.4)	5 (0.3)
No	77,632 (99.1)	3797 (98.6)	22,950 (99.1)	37,807 (99.1)	11,319 (99.0)	1759 (99.2)
Missing	475 (0.6)	31 (0.8)	132 (0.6)	230 (0.6)	72 (0.6)	10 (0.6)
**Uterine malformation, *n* (%)**						
Yes	223 (0.3)	8 (0.2)	74 (0.3)	110 (0.3)	27 (0.2)	4 (0.2)
No	77,668 (99.1)	3811 (99.0)	22,955 (99.1)	37,806 (99.1)	11,336 (99.1)	1760 (99.2)
Missing	475 (0.6)	31 (0.8)	132 (0.6)	230 (0.6)	72 (0.6)	10 (0.6)
**Malformation of urinary tract or genital organs, *n* (%)**						
Yes	30 (0.0)	0 (0.0)	11 (0.0)	15 (0.0)	4 (0.0)	0 (0.0)
No	77,861 (99.4)	3819 (99.2)	23,018 (99.4)	37,901 (99.4)	11,359 (99.3)	1764 (99.4)
Missing	475 (0.6)	31 (0.8)	132 (0.6)	230 (0.6)	72 (0.6)	10 (0.6)
**Currently smoking, *n* (%)**						
Yes	3104 (4.0)	209 (5.4)	999 (4.3)	1456 (3.8)	362 (3.2)	78 (4.4)
No	74,288 (94.8)	3584 (93.1)	21,877 (94.5)	36,217 (94.9)	10,934 (95.6)	1676 (94.5)
Missing	974 (1.2)	57 (1.5)	285 (1.2)	473 (1.2)	139 (1.2)	20 (1.1)
**Continue drinking, *n* (%)**						
Yes	7943 (10.1)	359 (9.3)	2304 (9.9)	3906 (10.2)	1175 (10.3)	199 (11.2)
No	69,624 (88.8)	3440 (89.4)	20,623 (89.0)	33,854 (88.7)	10,150 (88.8)	1557 (87.8)
Missing	799 (1.0)	51 (1.3)	234 (1.0)	386 (1.0)	110 (1.0)	18 (1.0)
**Use of any drug before 12 weeks of gestation** (methimazole, SSRI, antidepressant drug except for SSRI, antianxiety, sleeping pill, antipsychotic, valproic acid, antiepileptic except for valproic acid, lithium carbonate, and other psychoactive drug), *n* (%)	2154 (2.7)	133 (3.5)	672 (2.9)	1011 (2.7)	298 (2.6)	40 (2.3)
**Use of folic acid supplement before 12 weeks of gestation, *n* (%)**	25,604 (32.7)	1267 (32.9)	7493 (32.4)	12,543 (32.9)	3749 (32.8)	552 (31.1)
**New-onset HDP, *n* (%)**						
Yes	779 (1.0)	48 (1.2)	238 (1.0)	386 (1.0)	90 (0.8)	17 (1.0)
No	77,587 (99.0)	3802 (98.8)	22,923 (99.0)	37,760 (99.0)	11,345 (99.2)	1757 (99.0)
**GDM, *n* (%)**						
Yes	2132 (2.7)	153 (4.0)	682 (2.9)	976 (2.6)	272 (2.4)	49 (2.8)
No	76,092 (97.1)	3689 (95.8)	22,435 (96.9)	37,105 (97.3)	11,143 (97.4)	1720 (97.0)
Missing	142 (0.2)	8 (0.2)	44 (0.2)	65 (0.2)	20 (0.2)	5 (0.3)
**Maternal highest level of education, *n* (%)**						
<13 years	26,252 (33.5)	1518 (39.4)	7994 (34.5)	12,581 (33.0)	3536 (30.9)	623 (35.1)
≥13 years	51,185 (65.3)	2280 (59.2)	14,894 (64.3)	25,107 (65.8)	7779 (68.0)	1125 (63.4)
Missing	929 (1.2)	52 (1.4)	273 (1.2)	458 (1.2)	120 (1.0)	26 (1.5)
**Marital status, *n* (%)**						
Unmarried or divorced or widowed	3185 (4.1)	211 (5.5)	1020 (4.4)	1466 (3.8)	429 (3.8)	59 (3.3)
Married	74,426 (95.0)	3588 (93.2)	21,923 (94.7)	36,320 (95.2)	10,896 (95.3)	1699 (95.8)
Missing	755 (1.0)	51 (1.3)	218 (0.9)	360 (0.9)	110 (1.0)	16 (0.9)
**Annual household income (million,** **Japanese Yen), *n* (%)**						
<4	28,310 (36.1)	1551 (40.3)	8592 (37.1)	13,631 (35.7)	3899 (34.1)	637 (35.9)
4–5.99	36,476 (46.5)	1656 (43.0)	10,544 (45.5)	17,902 (46.9)	5552 (48.6)	822 (46.3)
≥6	8204 (10.5)	353 (9.2)	2371 (10.2)	4088 (10.7)	1211 (10.6)	181 (10.2)
Missing	5376 (6.9)	290 (7.5)	1654 (7.1)	2525 (6.6)	773 (6.8)	134 (7.6)
**Delivery week, weeks**	39.3 (1.5)	39.0 (1.7)	39.2 (1.6)	39.3 (1.5)	39.4 (1.5)	39.4 (1.5)
**Preterm delivery at <37 weeks of** **gestation, *n* (%)**						
Yes	3476 (4.4)	242 (6.3)	1174 (5.1)	1571 (4.1)	420 (3.7)	69 (3.9)
No	74,748 (95.4)	3600 (93.5)	21,943 (94.7)	36,510 (95.7)	10,995 (96.2)	1700 (95.8)
Missing	142 (0.2)	8 (0.2)	44 (0.2)	65 (0.2)	20 (0.2)	5 (0.3)
**Infant sex, *n* (%)**						
Male	40,117 (51.2)	1963 (51.0)	11,820 (51.0)	19,535 (51.2)	5884 (51.5)	915 (51.6)
Female	38,249 (48.8)	1887 (49.0)	11,341 (49.0)	18,611 (48.8)	5551 (48.5)	859 (48.4)
**Infant birth weight, g**	3026 (411)	2877 (425)	2933 (400)	3044 (395)	3171 (415)	3258 (425)
**Category of infant birth weight in** **percentiles, *n* (%)**						
SGA (<10th percentile)	5751 (7.3)	542 (14.1)	2418 (10.4)	2348 (6.2)	401 (3.5)	42 (2.4)
AGA (≥10th percentile and <90th percentile)	62,580 (79.9)	2975 (77.3)	18,625 (80.4)	31,036 (81.4)	8716 (76.2)	1228 (69.2)
LGA (≥90th percentile)	7708 (9.8)	221 (5.7)	1403 (6.1)	3652 (9.6)	1974 (17.3)	458 (25.8)
Missing	2327 (3.0)	112 (2.9)	715 (3.1)	1110 (2.9)	344 (3.0)	46 (2.6)
**Category of infant birth weight in grams, *n* (%)s**						
Low birth weight (<2500 g)	6176 (7.9)	570 (14.8)	2520 (10.9)	2526 (6.6)	497 (4.3)	63 (3.6)
Normal birth weight (≥2500 g and <4000 g)	71,333 (91.0)	3247 (84.3)	20,497 (88.5)	35,252 (92.4)	10,684 (93.4)	1653 (93.2)
Macrosomia (≥4000 g)	673 (0.9)	20 (0.5)	91 (0.4)	283 (0.7)	226 (2.0)	53 (3.0)
Missing	184 (0.2)	13 (0.3)	53 (0.2)	85 (0.2)	28 (0.2)	5 (0.3)

Data are expressed as mean (SD) or the number (percentage). ART, assisted reproductive technology; AGA, appropriate gestational age; BMI, body mass index; GDM, gestational diabetes mellitus; HDP, hypertensive disorders of pregnancy; LGA, large for gestational age; NGSP, National Glycohemoglobin Standardization Program; SD, standard deviation; SGA, small gestational age; SSRI, selective serotonin reuptake inhibitor.

**Table 2:** Prevalence of infant congenital malformations.

Infant Congenital Malformations	All Participants (*n* = 78,366)	Participants According to Maternal Birth Weight				
		<2500 g (*n* = 3850)	2500– 2999 g (*n* = 23,161)	3000– 3499 g (*n* = 38,146)	3500– 3999 g (*n* = 11,435)	≥4000 g (*n* = 1774)
**Nervous system, Cases (%)**	205 (0.26)	14 (0.36)	60 (0.26)	93 (0.24)	33 (0.29)	5 (0.28)
Anencephaly, Cases (%)	11 (0.01)	0 (0.00)	4 (0.02)	5 (0.01)	2 (0.02)	0 (0.00)
Encephalocele, Cases (%)	14 (0.02)	0 (0.00)	3 (0.01)	7 (0.02)	4 (0.03)	0 (0.00)
Microcephaly, Cases (%)	25 (0.03)	1 (0.03)	9 (0.04)	12 (0.03)	2 (0.02)	1 (0.06)
Hydrocephalus, Cases (%)	59 (0.08)	7 (0.18)	12 (0.05)	32 (0.08)	7 (0.06)	1 (0.06)
Holoprosencephaly, Cases (%)	20 (0.03)	2 (0.05)	6 (0.03)	7 (0.02)	5 (0.04)	0 (0.00)
Craniotabes, Cases (%)	58 (0.07)	4 (0.10)	19 (0.08)	22 (0.06)	12 (0.10)	1 (0.06)
Agenesis of corpus callosum, Cases (%)	17 (0.02)	1 (0.03)	4 (0.02)	8 (0.02)	4 (0.03)	0 (0.00)
Spina bifida, Cases (%)	24 (0.03)	2 (0.05)	7 (0.03)	12 (0.03)	1 (0.01)	2 (0.11)
**Eye, ear, and face, Cases (%)**	228 (0.29)	7 (0.18)	71 (0.31)	118 (0.31)	27 (0.24)	5 (0.28)
Eyelid coloboma, Cases (%)	12 (0.02)	1 (0.03)	3 (0.01)	8 (0.02)	0 (0.00)	0 (0.00)
Microphthalmia (anophthalmia), Cases (%)	13 (0.02)	0 (0.00)	2 (0.01)	9 (0.02)	1 (0.01)	1 (0.06)
Cataract, Cases (%)	28 (0.04)	1 (0.03)	8 (0.03)	15 (0.04)	4 (0.03)	0 (0.00)
Microtia, Cases (%)	39 (0.05)	0 (0.00)	13 (0.06)	24 (0.06)	2 (0.02)	0 (0.00)
Atresia of external auditory canal, Cases (%)	35 (0.04)	0 (0.00)	13 (0.06)	14 (0.04)	7 (0.06)	1 (0.06)
Cryptotia, Cases (%)	30 (0.04)	0 (0.00)	14 (0.06)	12 (0.03)	3 (0.03)	1 (0.06)
Low-set ear, Cases (%)	29 (0.04)	1 (0.03)	11 (0.05)	14 (0.04)	3 (0.03)	0 (0.00)
Hearing loss, Cases (%)	61 (0.08)	5 (0.13)	15 (0.06)	31 (0.08)	8 (0.07)	2 (0.11)
Facial cleft, Cases (%)	7 (0.01)	0 (0.00)	2 (0.01)	4 (0.01)	1 (0.01)	0 (0.00)
**Cleft lip and/or cleft palate, Cases (%)**	165 (0.21)	12 (0.31)	56 (0.24)	70 (0.18)	26 (0.23)	1 (0.06)
**Circulatory system, Cases (%)**	1141 (1.46)	72 (1.87)	331 (1.43)	516 (1.35)	187 (1.64)	35 (1.97)
Congenital heart disease, Cases (%)	1068 (1.36)	69 (1.79)	311 (1.34)	485 (1.27)	172 (1.50)	31 (1.75)
Arrhythmia, Cases (%)	84 (0.11)	3 (0.08)	25 (0.11)	34 (0.09)	17 (0.15)	5 (0.28)
**Respiratory system, Cases (%)**	32 (0.04)	1 (0.03)	7 (0.03)	17 (0.04)	6 (0.05)	1 (0.06)
Pulmonary sequestration, Cases (%)	8 (0.01)	0 (0.00)	2 (0.01)	3 (0.01)	3 (0.03)	0 (0.00)
CCAM, Cases (%)	14 (0.02)	1 (0.03)	3 (0.01)	7 (0.02)	3 (0.03)	0 (0.00)
Pulmonary hypoplasia, Cases (%)	14 (0.02)	0 (0.00)	3 (0.01)	9 (0.02)	1 (0.01)	1 (0.06)
**Digestive system, Cases (%)**	62 (0.08)	1 (0.03)	26 (0.11)	25 (0.07)	9 (0.08)	1 (0.06)
Oesophageal atresia, Cases (%)	6 (0.01)	0 (0.00)	4 (0.02)	1 (0.00)	1 (0.01)	0 (0.00)
Duodenal atresia, Cases (%)	11 (0.01)	0 (0.00)	3 (0.01)	6 (0.02)	1 (0.01)	1 (0.06)
Small intestinal atresia, Cases (%)	17 (0.02)	0 (0.00)	5 (0.02)	10 (0.03)	2 (0.02)	0 (0.00)
Imperforate anus (anorectal anomaly), Cases (%)	35 (0.04)	1 (0.03)	16 (0.07)	12 (0.03)	5 (0.04)	1 (0.06)
**Urinary system (CAKUT), Cases (%)**	271 (0.35)	17 (0.44)	66 (0.28)	135 (0.35)	39 (0.34)	14 (0.79)
Hydronephrosis, Cases (%)	229 (0.29)	13 (0.34)	61 (0.26)	109 (0.29)	33 (0.29)	13 (0.73)
Cystic renal anomalies, Cases (%)	31 (0.04)	2 (0.05)	4 (0.02)	19 (0.05)	5 (0.04)	1 (0.06)
Renal agenesis, Cases (%)	17 (0.02)	2 (0.05)	2 (0.01)	11 (0.03)	2 (0.02)	0 (0.00)
Bladder exstrophy/cloacal exstrophy, Cases (%)	2 (0.00)	0 (0.00)	0 (0.00)	2 (0.01)	0 (0.00)	0 (0.00)
**Genital organs in male infants, Cases/*n* (%)**	448/40,117 (1.12)	32/1963 (1.63)	139/11,820 (1.18)	194/19,535 (0.99)	74/5884 (1.26)	9/915 (0.98)
Hypospadias, Cases/*n* (%)	271/40,117 (0.68)	21/1963 (1.07)	85/11,820 (0.72)	118/19,535 (0.60)	40/5884 (0.68)	7/915 (0.77)
Cryptorchidism/nonpalpable testis, Cases/*n* (%)	266/40,117 (0.66)	18/1963 (0.92)	82/11,820 (0.69)	115/19,535 (0.59)	46/5884 (0.78)	5/915 (0.55)
Micropenis, Cases/*n* (%)	4/40,117 (0.01)	0/1963 (0.00)	1/11,820 (0.01)	2/19,535 (0.01)	1/5884 (0.02)	0/915 (0.00)
Bifid scrotum, Cases/*n* (%)	8/40,117 (0.02)	0/1963 (0.00)	3/11,820 (0.03)	3/19,535 (0.02)	2/5884 (0.03)	0/915 (0.00)
**Genital organs in female infants, Cases/*n* (%)**	11/38,249 (0.03)	0/1887 (0.00)	4/11,341 (0.04)	6/18,611 (0.03)	1/5551 (0.02)	0/859 (0.00)
Clitoral hypertrophy, Cases/*n* (%)	7/38,249 (0.02)	0/1887 (0.00)	4/11,341 (0.04)	3/18,611 (0.02)	0/5551 (0.00)	0/859 (0.00)
Abnormal vagina opening, Cases/*n* (%)	4/38,249 (0.01)	0/1887 (0.00)	0/11,341 (0.00)	3/18,611 (0.02)	1/5551 (0.02)	0/859 (0.00)
**Musculoskeletal system, Cases (%)**	289 (0.37)	8 (0.21)	94 (0.41)	135 (0.35)	40 (0.35)	12 (0.68)
Congenital diaphragmatic hernia, Cases (%)	29 (0.04)	1 (0.03)	9 (0.04)	13 (0.03)	6 (0.05)	0 (0.00)
Umbilical hernia, Cases (%)	32 (0.04)	3 (0.08)	8 (0.03)	14 (0.04)	4 (0.03)	3 (0.17)
Gastroschisis, Cases (%)	5 (0.01)	0 (0.00)	2 (0.01)	1 (0.00)	1 (0.01)	1 (0.06)
Scoliosis, Cases (%)	2 (0.00)	0 (0.00)	0 (0.00)	2 (0.01)	0 (0.00)	0 (0.00)
Limbs, *n* (%)	221 (0.28)	4 (0.10)	75 (0.32)	105 (0.28)	29 (0.25)	8 (0.45)
Polydactyly of upper limb, Cases (%)	79 (0.10)	2 (0.05)	31 (0.13)	38 (0.10)	6 (0.05)	2 (0.11)
Syndactyly of upper limb, Cases (%)	37 (0.05)	1 (0.03)	14 (0.06)	13 (0.03)	8 (0.07)	1 (0.06)
Brachydactyly of upper limb, Cases (%)	10 (0.01)	1 (0.03)	5 (0.02)	3 (0.01)	1 (0.01)	0 (0.00)
Cleft hand of upper limb, Cases (%)	6 (0.01)	0 (0.00)	3 (0.01)	3 (0.01)	0 (0.00)	0 (0.00)
Defect of upper limb, Cases (%)	2 (0.00)	0 (0.00)	1 (0.00)	1 (0.00)	0 (0.00)	0 (0.00)
Polydactyly of lower limb, Cases (%)	82 (0.10)	0 (0.00)	22 (0.09)	43 (0.11)	13 (0.11)	4 (0.23)
Syndactyly of lower limb, Cases (%)	88 (0.11)	2 (0.05)	21 (0.09)	45 (0.12)	16 (0.14)	4 (0.23)
Short toe, Cases (%)	2 (0.00)	0 (0.00)	1 (0.00)	1 (0.00)	0 (0.00)	0 (0.00)
Cleft foot, Cases (%)	6 (0.01)	0 (0.00)	3 (0.01)	3 (0.01)	0 (0.00)	0 (0.00)
Defect of lower limb, Cases (%)	3 (0.00)	0 (0.00)	1 (0.00)	2 (0.01)	0 (0.00)	0 (0.00)
**Skin, Cases (%)**	610 (0.78)	43 (1.12)	174 (0.75)	292 (0.77)	89 (0.78)	12 (0.68)
Angioma, Cases (%)	600 (0.77)	43 (1.12)	172 (0.74)	286 (0.75)	87 (0.76)	12 (0.68)
Epidermolysis bullosa hereditaria/ incontinence of pigment, Cases (%)	10 (0.01)	0 (0.00)	2 (0.01)	6 (0.02)	2 (0.02)	0 (0.00)
**Inguinal Hernia, Cases (%)**	382 (0.49)	31 (0.81)	106 (0.46)	175 (0.46)	59 (0.52)	11 (0.62)

Data are expressed as a number (percentage). Abbreviations: CAKUT, congenital anomalies of the kidney and urinary tract; CCAM, congenital cystic adenomatoid malformation.

**Table 3:** Association between MBW and prevalence of infant congenital malformations.

Infant Congenital Malformations	Maternal Birth Weight				
	<2500 g (*n* = 3850)	2500– 2999 g (*n* = 23,161)	3000– 3499 g (*n* = 38,146)	3500– 3999 g (*n* = 11,435)	≥4000 g (*n* = 1774)
**Nervous system**					
Cases (%)	14 (0.36)	60 (0.26)	93 (0.24)	33 (0.29)	5 (0.28)
Model 1, Crude OR (95% CI) ^a^	1.538 (0.848–2.590)	1.066 (0.768–1.469)	Reference	1.196 (0.794–1.756)	1.256 (0.470–2.724)
Model 2, Adjusted OR (95% CI) ^a, b^	1.242 (0.947–1.628)	1.029 (0.878–1.206)	Reference	1.098 (0.905–1.332)	1.144 (0.751–1.744)
**Eye, ear, and face**					
Cases (%)	7 (0.18)	71 (0.31)	118 (0.31)	27 (0.24)	5 (0.28)
Model 1, Crude OR (95% CI) ^a^	0.626 (0.273–1.222)	0.994 (0.738–1.329)	Reference	0.774 (0.501–1.153)	0.997 (0.372–2.134)
Model 2, Adjusted OR (95% CI) ^a, b^	0.792 (0.552–1.136)	0.998 (0.864–1.153)	Reference	0.875 (0.714–1.073)	0.987 (0.649–1.502)
**Cleft lip and/or cleft palate**					
Cases (%)	12 (0.31)	56 (0.24)	70 (0.18)	26 (0.23)	1 (0.06)
Model 1, Crude OR (95% CI) ^a^	1.759 (0.918–3.096)	1.321 (0.927–1.872)	Reference	1.254 (0.789–1.936)	0.457 (0.052–1.666)
Model 2, Adjusted OR (95% CI) ^a, b^	1.331 (0.994–1.784)	1.154 (0.973–1.368)	Reference	1.114 (0.896–1.385)	0.668 (0.304–1.466)
**Circulatory system**					
**Congenital heart diseases**					
Cases (%)	69 (1.79)	311 (1.34)	485 (1.27)	172 (1.50)	31 (1.75)
Model 1, Crude OR (95% CI)	1.417 (1.099–1.828)	1.057 (0.916–1.220)	Reference	1.186 (0.995–1.413)	1.381 (0.958–1.992)
Model 2, Adjusted OR (95% CI) ^b^	1.388 (1.075–1.792)	1.050 (0.909–1.212)	Reference	1.179 (0.989–1.405)	1.370 (0.949–1.977)
**Arrhythmia**					
Cases (%)	3 (0.08)	25 (0.11)	34 (0.09)	17 (0.15)	5 (0.28)
Model 1, Crude OR (95% CI) ^a^	1.005 (0.273–2.639)	1.218 (0.723–2.024)	Reference	1.693 (0.931–2.969)	3.435 (1.240–7.793)
Model 2, Adjusted OR (95% CI) ^a, b^	1.029 (0.613–1.729)	1.123 (0.881–1.432)	Reference	1.284 (0.977–1.688)	1.775 (1.157–2.725)
**Urinary system (CAKUT)**					
Cases (%)	17 (0.44)	66 (0.28)	135 (0.35)	39 (0.34)	14 (0.79)
Model 1, Crude OR (95% CI)	1.249 (0.753–2.070)	0.805 (0.599–1.081)	Reference	0.964 (0.674–1.377)	2.240 (1.289–3.891)
** Model 2, Adjusted OR (95% CI) ^b^**	1.254 (0.756–2.083)	0.809 (0.602–1.087)	Reference	0.957 (0.669–1.368)	2.194 (1.261–3.819)
**Genital organs in male infants, *n***	*n* = 1963	*n* = 11,820	*n* = 19,535	*n* = 5884	*n* = 915
Cases (%)	32 (1.63)	139 (1.18)	194 (0.99)	74 (1.26)	9 (0.98)
Model 1, Crude OR (95% CI)	1.652 (1.133–2.408)	1.186 (0.953–1.477)	Reference	1.270 (0.970–1.662)	0.990 (0.506–1.939)
Model 2, Adjusted OR (95% CI) ^b^	1.648 (1.130–2.405)	1.189 (0.954–1.481)	Reference	1.264 (0.965–1.655)	0.974 (0.497–1.907)
**Hypospadias in male infants**					
Cases (%)	21 (1.07)	85 (0.72)	118 (0.60)	40 (0.68)	7 (0.77)
Model 1, Crude OR (95% CI)	1.779 (1.116–2.837)	1.192 (0.901–1.577)	Reference	1.126 (0.786–1.614)	1.269 (0.590–2.727)
Model 2, Adjusted OR (95% CI) ^b^	1.804 (1.130–2.881)	1.199 (0.906–1.587)	Reference	1.115 (0.778–1.599)	1.250 (0.581–2.691)
**Cryptorchidism/nonpalpable** **testis in male infants**					
Cases (%)	18 (0.92)	82 (0.69)	115 (0.59)	46 (0.78)	5 (0.55)
Model 1, Crude OR (95% CI)	1.563 (0.949–2.574)	1.180 (0.888–1.567)	Reference	1.331 (0.944–1.875)	0.928 (0.378–2.277)
Model 2, Adjusted OR (95% CI) ^b^	1.515 (0.919–2.498)	1.170 (0.880–1.556)	Reference	1.332 (0.945–1.879)	0.912 (0.371–2.240)
**Musculoskeletal system**					
**Limbs**					
Cases (%)	4 (0.10)	75 (0.32)	105 (0.28)	29 (0.25)	8 (0.45)
Model 1, Crude OR (95% CI)	0.377 (0.139–1.023)	1.177 (0.875–1.584)	Reference	0.921 (0.610–1.390)	1.641 (0.799–3.373)
Model 2, Adjusted OR (95% CI) ^b^	0.370 (0.136–1.007)	1.168 (0.868–1.573)	Reference	0.912 (0.604–1.378)	1.634 (0.794–3.363)
**Skin**					
**Angioma**					
Cases (%)	43 (1.12)	174 (0.75)	292 (0.77)	89 (0.78)	12 (0.68)
Model 1, Crude OR (95% CI)	1.495 (1.083–2.064)	0.990 (0.819–1.197)	Reference	1.015 (0.798–1.291)	0.902 (0.505–1.609)
Model 2, Adjusted OR (95% CI) ^b^	1.491 (1.079–2.059)	0.993 (0.821–1.201)	Reference	1.004 (0.789–1.278)	0.882 (0.494–1.576)
**Inguinal hernia**					
Cases (%)	31 (0.81)	106 (0.46)	175 (0.46)	59 (0.52)	11 (0.62)
Model 1, Crude OR (95% CI)	1.761 (1.200–2.584)	0.998 (0.783–1.270)	Reference	1.125 (0.837–1.513)	1.354 (0.735–2.494)
Model 2, Adjusted OR (95% CI) ^b^	1.746 (1.189–2.565)	0.997 (0.783–1.271)	Reference	1.119 (0.832–1.504)	1.344 (0.729–2.479)

^a^—Firth logistic regression model was applied. ^b^—Adjusted for maternal age in the MT1 questionnaire, pre-pregnancy BMI, conception method, parity (primipara or not), history of mental illness, history of kidney disease, history of congenital heart disease, history of uterine malformation and/or urogenital malformation, smoking status, alcohol consumption, marital status, education level, annual income, use of any drug before 12 weeks of gestation (methimazole, SSRI, antidepressant drug except for SSRI, antianxiety, sleeping pill, antipsychotic, valproic acid, antiepileptic except for valproic acid, lithium carbonate, and other psychoactive drug), use of folic acid supplement at <12 weeks of gestation, HbA1c level at <24 weeks of gestation, and infant sex. In the analysis of the association between maternal birth weight and genital organs in male infants, infant sex was not included in the model. Abbreviations: BMI, body mass index; CAKUT, congenital anomalies of the kidney and urinary tract; CI, confidence interval; HbA1c, glycosylated haemoglobin; OR, odds ratio; SSRI, selective serotonin reuptake inhibitor.

**Table 4:** Association between MBW and prevalence of infant congenital malformations stratified according to infant sex.

Infant Congenital Malformations	Maternal Birth Weight				
	<2500 g	2500–2999 g	3000–3499 g	3500–3999 g	≥4000 g
**The number of male and female** **infants**					
Male infants, *n*	1963	11,820	19,535	5884	915
Female infants, *n*	1887	11,341	18,611	5551	859
**Nervous system**					
** Male infants**					
Cases (%)	9 (0.46)	30 (0.25)	55 (0.28)	17 (0.29)	2 (0.22)
Model 1, Crude OR (95% CI) ^a^	1.706 (0.803–3.240)	0.908 (0.577–1.402)	Reference	1.047 (0.594–1.755)	0.961 (0.199–2.804)
Model 2, Adjusted OR (95% CI) ^a, b^	1.306 (0.936–1.821)	0.952 (0.769–1.178)	Reference	1.024 (0.790–1.328)	0.988 (0.536–1.822)
** Female infants**					
Cases (%)	5 (0.26)	30 (0.26)	38 (0.20)	16 (0.29)	3 (0.35)
Model 1, Crude OR (95% CI) ^a^	1.409 (0.511–3.172)	1.301 (0.803–2.089)	Reference	1.438 (0.786–2.516)	1.971 (0.537–5.149)
Model 2, Adjusted OR (95% CI) ^a, b^	1.192 (0.780–1.823)	1.138 (0.906–1.428)	Reference	1.208 (0.916–1.592)	1.437 (0.850–2.428)
**Eye, ear, and face**					
** Male infants**					
Cases (%)	2 (0.10)	45 (0.38)	68 (0.35)	12 (0.20)	3 (0.33)
Model 1, Crude OR (95% CI) ^a^	0.363 (0.075–1.048)	1.098 (0.750–1.593)	Reference	0.605 (0.315–1.066)	1.090 (0.301–2.768)
Model 2, Adjusted OR (95% CI) ^a, b^	0.599 (0.326–1.101)	1.048 (0.874–1.257)	Reference	0.777 (0.580–1.040)	1.041 (0.618–1.753)
** Female infants**					
Cases (%)	5 (0.26)	26 (0.23)	50 (0.27)	15 (0.27)	2 (0.23)
Model 1, Crude OR (95% CI) ^a^	1.074 (0.393–2.374)	0.861 (0.530–1.365)	Reference	1.029 (0.563–1.775)	1.072 (0.221–3.141)
Model 2, Adjusted OR (95% CI) ^a, b^	1.047 (0.687–1.591)	0.932 (0.744–1.168)	Reference	1.015 (0.773–1.334)	1.014 (0.552–1.865)
**Cleft lip and/or cleft palate**					
** Male infants**					
Cases (%)	9 (0.46)	33 (0.28)	43 (0.22)	14 (0.24)	1 (0.11)
Model 1, Crude OR (95% CI) ^a^	2.179 (1.014–4.208)	1.274 (0.806–1.995)	Reference	1.107 (0.590–1.957)	0.735 (0.083–2.729)
Model 2, Adjusted OR (95% CI) ^a, b^	1.473 (1.052–2.064)	1.132 (0.912–1.406)	Reference	1.050 (0.789–1.396)	0.845 (0.386–1.846)
** Female infants**					
Cases (%)	3 (0.16)	23 (0.20)	27 (0.15)	12 (0.22)	0 (0.00)
Model 1, Crude OR (95% CI) ^a^	1.255 (0.338–3.358)	1.403 (0.803–2.433)	Reference	1.525 (0.754–2.914)	0.393 (0.003–2.802)
Model 2, Adjusted OR (95% CI) ^a, b^	1.137 (0.678–1.906)	1.183 (0.913–1.533)	Reference	1.219 (0.890–1.671)	0.626 (0.169–2.314)
**Circulatory system**					
** Congenital heart diseases**					
** Male infants**					
Cases (%)	34 (1.73)	144 (1.22)	206 (1.05)	82 (1.39)	17 (1.86)
Model 1, Crude OR (95% CI)	1.654 (1.147–2.384)	1.157 (0.934–1.434)	Reference	1.326 (1.025–1.716)	1.776 (1.078–2.926)
Model 2, Adjusted OR (95% CI) ^b^	1.615 (1.119–2.332)	1.154 (0.931–1.430)	Reference	1.317 (1.017–1.704)	1.745 (1.058–2.877)
** Female infants**					
Cases (%)	35 (1.85)	167 (1.47)	279 (1.50)	90 (1.62)	14 (1.63)
Model 1, Crude OR (95% CI) ^a^	1.257 (0.869–1.762)	0.983 (0.809–1.191)	Reference	1.087 (0.852–1.374)	1.125 (0.631–1.845)
Model 2, Adjusted OR (95% CI) ^a, b^	1.113 (0.934–1.327)	0.988 (0.897–1.087)	Reference	1.041 (0.925–1.173)	1.062 (0.815–1.383)
** Arrhythmia**					
** Male infants**					
Cases (%)	0 (0.00)	13 (0.11)	15 (0.08)	9 (0.15)	2 (0.22)
Model 1, Crude OR (95% CI) ^a^	0.321 (0.003–2.379)	1.440 (0.684–2.994)	Reference	2.036 (0.874–4.500)	3.447 (0.682–11.141)
Model 2, Adjusted OR (95% CI) ^a, b^	0.558 (0.169–1.947)	1.190 (0.855–1.656)	Reference	1.427 (0.988–2.059)	1.840 (0.999–3.387)
** Female infants**					
Cases (%)	3 (0.16)	12 (0.11)	19 (0.10)	8 (0.14)	3 (0.35)
Model 1, Crude OR (95% CI) ^a^	1.771 (0.469–4.912)	1.052 (0.504–2.117)	Reference	1.462 (0.618–3.172)	3.897 (1.031–10.823)
Model 2, Adjusted OR (95% CI) ^a, b^	1.394 (0.833–2.333)	1.056 (0.763–1.462)	Reference	1.171 (0.808–1.698)	1.788 (1.047–3.052)
**Urinary system (CAKUT)**					
** Male infants**					
Cases (%)	8 (0.41)	51 (0.43)	102 (0.52)	31 (0.53)	12 (1.31)
Model 1, Crude OR (95% CI)	0.780 (0.379–1.603)	0.826 (0.589–1.156)	Reference	1.009 (0.674–1.510)	2.532 (1.387–4.622)
Model 2, Adjusted OR (95% CI) ^b^	0.808 (0.392–1.664)	0.837 (0.597–1.173)	Reference	0.996 (0.665–1.491)	2.470 (1.350–4.517)
** Female infants**					
Cases (%)	9 (0.48)	15 (0.13)	33 (0.18)	8 (0.14)	2 (0.23)
Model 1, Crude OR (95% CI) ^a^	2.805 (1.287–5.547)	0.759 (0.404–1.362)	Reference	0.850 (0.374–1.723)	1.617 (0.330–4.845)
Model 2, Adjusted OR (95% CI) ^a, b^	1.619 (1.154–2.273)	0.860 (0.647–1.141)	Reference	0.926 (0.649–1.321)	1.267 (0.691–2.322)
**Musculoskeletal system**					
** Limbs**					
** Male infants**					
Cases (%)	1 (0.05)	46 (0.39)	56 (0.29)	16 (0.27)	4 (0.44)
Model 1, Crude OR (95% CI)	0.178 (0.025–1.281)	1.359 (0.919–2.009)	Reference	0.948 (0.544–1.654)	1.527 (0.553–4.221)
Model 2, Adjusted OR (95% CI) ^b^	0.176 (0.024–1.270)	1.354 (0.915–2.003)	Reference	0.922 (0.528–1.609)	1.514 (0.547–4.194)
** Female infants**					
Cases (%)	3 (0.16)	29 (0.26)	49 (0.26)	13 (0.23)	4 (0.47)
Model 1, Crude OR (95% CI) ^a^	0.696 (0.191–1.791)	0.978 (0.613–1.533)	Reference	0.914 (0.481–1.621)	1.974 (0.642–4.658)
Model 2, Adjusted OR (95% CI) ^a, b^	0.822 (0.491–1.375)	0.981 (0.788–1.221)	Reference	0.960 (0.719–1.281)	1.404 (0.882–2.233)
**Skin**					
** Angioma**					
** Male infants**					
Cases (%)	16 (0.82)	72 (0.61)	99 (0.51)	35 (0.59)	5 (0.55)
Model 1, Crude OR (95% CI) ^a^	1.655 (0.946–2.713)	1.205 (0.887–1.630)	Reference	1.186 (0.797–1.723)	1.180 (0.439–2.540)
Model 2, Adjusted OR (95% CI) ^b^	1.292 (0.999–1.670)	1.101 (0.948–1.278)	Reference	1.082 (0.896–1.307)	1.082 (0.709–1.651)
** Female infants**					
Cases (%)	27 (1.43)	100 (0.88)	187 (1.00)	52 (0.94)	7 (0.81)
Model 1, Crude OR (95% CI) ^a^	1.452 (0.979–2.134)	0.878 (0.686–1.118)	Reference	0.938 (0.683–1.266)	0.864 (0.379–1.675)
Model 2, Adjusted OR (95% CI) ^a, b^	1.196 (0.980–1.461)	0.939 (0.832–1.059)	Reference	0.963 (0.827–1.122)	0.914 (0.636–1.314)
**Inguinal hernia**					
** Male infants**					
Cases (%)	23 (1.17)	56 (0.47)	106 (0.54)	30 (0.51)	9 (0.98)
Model 1, Crude OR (95% CI) ^a^	2.209 (1.378–3.397)	0.876 (0.630–1.205)	Reference	0.950 (0.624–1.404)	1.912 (0.916–3.530)
Model 2, Adjusted OR (95% CI) ^a, b^	1.484 (1.189–1.851)	0.938 (0.800–1.099)	Reference	0.972 (0.798–1.186)	1.373 (0.989–1.907)
** Female infants**					
Cases (%)	8 (0.42)	50 (0.44)	69 (0.37)	29 (0.52)	2 (0.23)
Model 1, Crude OR (95% CI) ^a^	1.207 (0.548–2.326)	1.193 (0.826–1.711)	Reference	1.425 (0.912–2.171)	0.778 (0.161–2.252)
Model 2, Adjusted OR (95% CI) ^a, b^	1.092 (0.772–1.543)	1.091 (0.914–1.302)	Reference	1.195 (0.968–1.475)	0.877 (0.474–1.622)

^a^—Firth logistic regression model was applied. ^b^—Adjusted for maternal age at the MT1 questionnaire, pre-pregnancy BMI, conception method, parity (primipara or not), history of mental illness, history of kidney disease, history of congenital heart disease, history of uterine malformation and/or urogenital malformation, smoking status, alcohol consumption, marital status, education level, annual income, use of any drug before 12 weeks of gestation (methimazole, SSRI, antidepressant drug except for SSRI, antianxiety, sleeping pill, antipsychotic, valproic acid, antiepileptic except for valproic acid, lithium carbonate, and other psychoactive drug), use of folic acid supplement at <12 weeks of gestation, and HbA1c level at <24 weeks of gestation. Abbreviations: BMI, body mass index; CAKUT, congenital anomalies of the kidney and urinary tract; CI, confidence interval; HbA1c, glycosylated haemoglobin; OR, odds ratio; SSRI, selective serotonin reuptake inhibitor.

## Data Availability

Data are unsuitable for public deposition because of ethical restrictions and the legal framework of Japan. It is prohibited by the Act on the Protection of Personal Information (Act No. 57 of 30 May 2003 amended on 9 September 2015) to publicly deposit data containing personal information. Ethical guidelines for medical and health research involving human participants enforced by the Japan Ministry of Education, Culture, Sports, Science and Technology and the Ministry of Health, Labour and Welfare also restrict the open sharing of epidemiological data. All enquiries about access to data should be sent to jecs-en@nies.go.jp. The person responsible for handling enquiries sent to this e-mail address is Dr. Shoji F. Nakayama, JECS Programme Office, National Institute for Environmental Studies. Regarding data for the paper publication, http://www.env.go.jp/chemi/ceh/en/index.html (accessed 9 August 2021).

## Appendix A

Members of the JECS Group as of 2023: Michihiro Kamijima (principal investigator, Nagoya City University, Nagoya, Japan), Shin Yamazaki (National Institute for Environmental Studies, Tsukuba, Japan), Yukihiro Ohya (National Center for Child Health and Development, Tokyo, Japan), Reiko Kishi (Hokkaido University, Sapporo, Japan), Nobuo Yaegashi (Tohoku University, Sendai, Japan), Koichi Hashimoto (Fukushima Medical University, Fukushima, Japan), Chisato Mori (Chiba University, Chiba, Japan), Shuichi Ito (Yokohama City University, Yokohama, Japan), Zentaro Yamagata (University of Yamanashi, Chuo, Japan), Hidekuni Inadera (University of Toyama, Toyama, Japan), Takeo Nakayama (Kyoto University, Kyoto, Japan), Tomotaka Sobue (Osaka University, Suita, Japan), Masayuki Shima (Hyogo Medical University, Nishinomiya, Japan), Seiji Kageyama (Tottori University, Yonago, Japan), Narufumi Suganuma (Kochi University, Nankoku, Japan), Shoichi Ohga (Kyushu University, Fukuoka, Japan), and Takahiko Katoh (Kumamoto University, Kumamoto, Japan).
